# Airway IL-1β associates with IL-5 production following dust mite allergen inhalation in humans

**DOI:** 10.1186/s12931-021-01903-9

**Published:** 2021-12-07

**Authors:** Allison J. Burbank, Stephen A. Schworer, Amika Sood, Martha Almond, Kelly Chason, Nathan Bean, Haibo Zhou, Michelle L. Hernandez

**Affiliations:** 1 Children’s Research Institute, University of North Carolina at Chapel Hill, 116 Manning Dr, CB#7231, Chapel Hill, NC 27599-7310 USA; 2 Division of Pediatric Allergy and Immunology, University of North Carolina, Chapel Hill, USA; 3 Arkansas Children’s Hospital, University of Arkansas for Medical Sciences, Little Rock, AR USA; 4 Center for Environmental Medicine, Asthma and Lung Biology, University of North Carolina, Chapel Hill, USA; 5 Department of Biostatistics, Gillings School of Global Public Health, University of North Carolina, Chapel Hill, NC USA

**Keywords:** Asthma, Allergic, Interleukin-1, T2-dominant asthma, Dust mite

## Abstract

**Background:**

Preclinical studies implicate interleukin (IL)-1β as a key mediator of asthma and have shown the efficacy of IL-1 antagonism for treatment of allergic airway inflammation; human studies in this area are lacking.

**Objectives:**

Our aim was to study the relationship of airway IL-1β to features of acute allergen-induced asthma exacerbation in humans.

**Methods:**

Dust mite-allergic adults with mild asthma underwent inhalation challenge with *Dermatophagoides farinae*. Fractional exhaled nitric oxide (FeNO), induced sputum and peripheral blood samples were obtained pre- and 24 h post-challenge. Spirometry was performed before and throughout the challenge at 10-min intervals, and allergen responsiveness was defined by a 20% fall in Forced Expiratory Volume in 1 s (FEV_1_). Sputum samples were analyzed for inflammatory cells, cytokines and chemokines. Multiple linear regression was employed to test the association between sputum IL-1β concentration and biomarkers of T helper type 2 (T2)-dominant inflammation.

**Results:**

Fourteen volunteers underwent inhaled allergen challenge. Allergen responsive volunteers showed a greater positive change in IL-1β in sputum following allergen challenge compared to non-responders. Higher pre-challenge sputum IL-1β was associated with greater increase in sputum IL-5 (p = 0.004), sputum eosinophils (p = 0.001) and blood IL-5 (p = 0.003) following allergen challenge. Allergen-induced sputum IL-1β production was significantly associated with sputum and blood IL-5 (p < 0.001 and p = 0.007, respectively), sputum IL-4 (p = 0.001), IL-13 (p = 0.026), eosinophils (p = 0.008) and FeNO (p = 0.03).

**Conclusions:**

The positive association between production of IL-1β and biomarkers of T2 inflammation, particularly IL-5, in humans is consistent with work in animal models that demonstrates a link between IL-1β and the pathophysiology of allergic asthma. The role of IL-1β in human asthma warrants further study.

## Background

Increased recognition of complex asthma endotypes has prompted new efforts to understand the pathophysiology of severe and difficult-to-control asthma and to identify new treatment targets. Interleukin (IL)-1 is a crucial mediator of inflammation with a vast array of functions in the immune system, and preclinical data suggest it is an important mediator of asthma pathogenesis. In the lung, IL-1β induces transcription of pro-inflammatory genes that impact granulocyte recruitment, airway hyperreactivity, and mucous secretion [1, 2]. Humans with asthma showed greater IL-1β expression in bronchial epithelial cells and macrophages compared to non-asthmatics [3]. IL-1 antagonism was associated with reduction in airway hyperreactivity and eosinophil recruitment in a mouse model of T helper type 2 (T2)-predominant asthma [4] and reduction in the delayed response to intradermal allergen administration in humans [5], suggesting a role for IL-1β in the generation of allergic inflammation. Purported mechanisms for this activity include IL-1β induction of T2 cytokine production (e.g. IL-4, IL-5 and IL-13) by T helper cells and increased expression of cellular adhesion molecules necessary for eosinophil recruitment [6]. There is a paucity of human studies describing the relationship of airway IL-1β to features of allergic airway inflammation. We conducted inhaled allergen challenge in 14 adults with mild allergic asthma to investigate whether airway IL-1β is associated with airway and systemic T2 inflammation in humans.

## Methods

Adults with mild intermittent asthma and sensitization to house dust mite (*Dermatophagoides farinae*, DF) underwent inhalation challenge with serial dilutions of DF extract (Stallergenes Greer®, Lenoir, NC) prepared in sterile saline using a protocol previously employed by our center [7]. Participants who experienced a ≥ 20% fall in forced expiratory volume in 1 s (FEV1) from baseline with inhaled allergen challenge were considered DF-responsive and observed for 10 h with spirometry performed at regular intervals. Spirometry, fractional exhaled nitric oxide (FeNO) measurements (NIOX Vero®, Circassia Pharmaceuticals, Inc, Morrisville, NC), blood and sputum samples were collected. Cytokines from sputum supernatants and serum were measured using multiplex immunoassays (Meso Scale Discovery®, Gaithersburg, MD). Within-group and between-group comparisons were made using Wilcoxon signed rank test and Mann–Whitney U test, respectively. Multiple linear regression was employed to test the association between sputum IL-1β concentration and inflammatory mediators of interest while adjusting for age and sex. Regression assumptions were reasonably satisfied, and no data transformations were necessary. Written informed consent was obtained from all participants, and the study was approved by the University of North Carolina Institutional Review Board. The study is listed on ClinicalTrials.gov (NCT03049111).

## Results

**Fig. 1:**
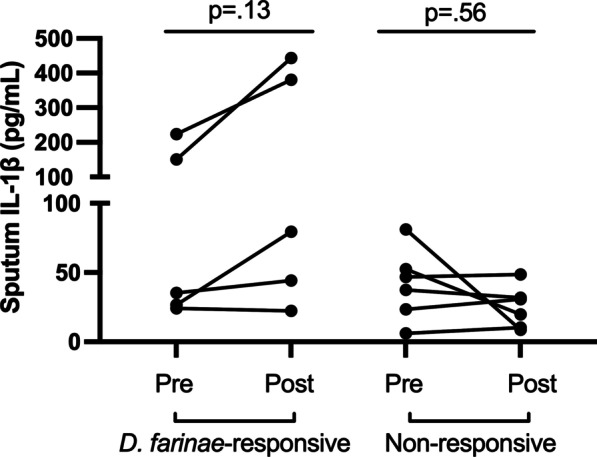
Sputum IL-1β in *D. farinae*-responsive and non-responsive asthmatics. Fourteen participants underwent inhalation challenge with *D. farinae*, with sputum samples collected before and 24 h after challenge. Paired sputum samples were available for 11 of the participants, including 5 *D. farinae-*responsive and 6 non-responsive individuals. Pre and post-challenge sputum IL-1β concentrations were compared within groups using Wilcoxon signed rank test

Fourteen non-smoking volunteers with mild intermittent allergic asthma and sensitization to *D. farinae* underwent inhaled allergen challenge. The study population was 43% female with mean age of 27 (± 7, SD) years and BMI 27 (± 6) kg/m^2^. Eight (57%) volunteers were DF-responsive, and 6 participants were non-responsive. Pre-challenge sputum IL-1β concentrations were similar between DF-responsive and non-responsive participants. While sputum IL-1β did not significantly increase from pre-challenge levels in either group following allergen challenge (Fig. 1), the DF-responsive group showed a significantly greater positive change in sputum IL-1β following allergen challenge compared to non-responders [mean (SD) Δ sputum IL-1β = 102 (± 123.7) pg/mL (DF-responsive) v. -16.3 (± 31.1) pg/mL (non-responsive), p = 0.03].

**Fig. 2:**
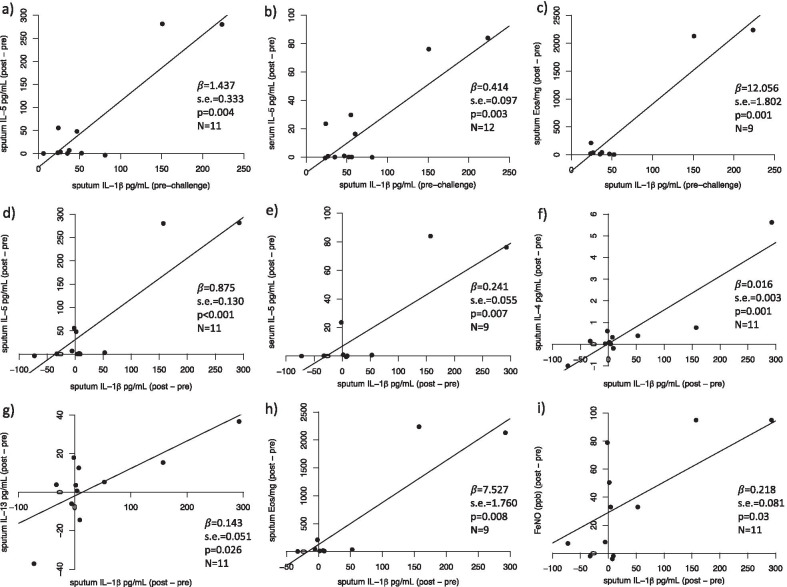
Sputum IL-1β concentration vs T2 inflammatory mediators. Multiple linear regression analysis was used to estimate the association of T2 inflammatory mediators to pre-challenge sputum IL-1β (**a**–**c**) and to allergen-induced change in sputum IL-1β (**d**–**i**), after adjusting for age and sex. In each plot, the regression line is fit by fixing age as the mean age of all participants and sex as the proportion of males. *β* effect estimate; *s.e.* standard error

In order to examine the relationship between sputum IL-1β and T2-dominant inflammation in response to allergen challenge, we performed regression analysis controlling for age and sex. Higher pre-challenge sputum IL-1β concentrations were associated with greater allergen-induced IL-5 in sputum and blood and sputum eosinophils (Fig. 2a–c). Greater allergen-induced rise in sputum IL-1β concentration was significantly associated with higher IL-5 in sputum and blood, higher sputum IL-4, IL-13 and eosinophils, and higher FeNO (Fig. 2d–i).

## Discussion

Airway inflammation in allergic asthma, the most common asthma endotype, is dominated by T2 cytokines including IL-4, IL-5 and IL-13. IL-5 is a critical mediator of eosinophil survival and recruitment to the airways in asthma. We detected a significant positive association between allergen-induced airway production of IL-1β and both airway and systemic production of IL-5 in adults with mild asthma. Additionally, we found that individuals with higher pre-challenge sputum IL-1β generated higher airway concentrations of IL-5, which could suggest that baseline IL-1β has a priming effect on T2-dominant airway inflammation. The impact may not be restricted to the airways, as we also showed that volunteers with higher pre-challenge sputum IL-1β displayed greater systemic production of IL-5 following allergen inhalation.

An imbalance of pro-inflammatory IL-1β and anti-inflammatory IL-1 receptor antagonist (IL-1RA) is a suggested mechanism for development of inflammatory diseases, including asthma [8]. Using a lipopolysaccharide (LPS) challenge model in humans, we previously reported that the synthetic IL-1 receptor antagonist, anakinra, reduced LPS-induced airway granulocyte recruitment and IL-1β concentrations compared to placebo [9]**.** The relationship detected between IL-1β and allergen-induced T2 biomarker responses in this study suggests anakinra could be studied for potential efficacy in allergic asthma as well.

The complexity and risks of inhaled allergen challenge procedures combined with restrictions put into place during the COVID-19 pandemic limited the size of our sample to a small number of otherwise healthy adults with mild intermittent allergic asthma. As such, the effects of allergen challenge on IL-1β and T2 cytokine production may not be reflective of the effects seen in patients with more severe forms of asthma.

## Conclusions

Prior work in animal models suggests that IL-1 is an important mediator of T2-dominant asthma. Though our study design does not allow for assessment of causality, our findings do demonstrate a positive association between IL-1β and IL-5 production in humans with allergic asthma that warrants further study.

## Data Availability

The datasets used and/or analyzed during the current study will be made available from the corresponding author on reasonable request.

## Abbreviations

IL: Interleukin
DF: *Dermatophagoides farinae*
FeNO: Fractional exhaled nitric oxide
FEV1: Forced expiratory volume in 1 s
T2 cells: T helper type 2
IL-1RA: Interleukin-1 receptor antagonist
LPS: Lipopolysaccharide
